# Factors related to self-care in Korean patients with tuberculosis: A systematic review and meta-analysis

**DOI:** 10.1097/MD.0000000000039920

**Published:** 2024-09-27

**Authors:** Hye Kyung Lee, Go Un Lee

**Affiliations:** a Department of Nursing, Kongju National University, Gongju, Republic of Korea.

**Keywords:** Korean patients, meta-analysis, self-care, social support, systematic review, tuberculosis

## Abstract

**Background:**

Tuberculosis (TB) remains a major public health challenge in South Korea which has one of the highest TB incidence rates among the Organisation for Economic Co-operation and Development (OECD) countries. Effective self-care, including medication adherence and regular hospital visits, is crucial for successful TB treatment and the prevention of drug resistance. TB self-care in South Korea is influenced by cultural, social, and systemic factors. This study aimed to systematically review and conduct a meta-analysis of factors influencing self-care among Korean patients with TB, providing evidence-based insights for developing effective self-care promotion programs.

**Methods:**

A systematic review and meta-analysis were conducted according to the Preferred Reporting Items for Systematic Reviews and Meta-Analyses (PRISMA) guidelines, focusing on quantitative studies published since 2000 involving Korean patients with TB. Twenty studies were included in the final analysis, and 44 factors related to self-care were categorized into sociodemographic, TB-related, psychological, environmental, and educational characteristics. Effect sizes were calculated using Comprehensive Meta-Analysis (CMA) 4.0, with the assessment of heterogeneity and publication bias.

**Results:**

The meta-analysis ranked the effect sizes of the different characteristic categories as follows: environmental > educational > psychological. Among the individual factors, social support had the greatest influence on self-care, followed by quality of life, self-efficacy, nonfamily support, family support, and perceived health benefits. These findings underscore the critical role of sustained social support from the community, medical staff, and family in enhancing self-care among TB patients.

**Conclusion:**

Effective self-care strategies for patients with TB should focus on interventions that strengthen the environmental, educational, and psychological aspects. These findings suggest that similar approaches can be applied in other countries facing comparable healthcare challenges. This study acknowledges limitations including potential publication bias and the exclusion of older studies and non-Korean patient studies, highlighting the need for further research across diverse settings and populations.

## 1. Introduction

In 2021, the World Health Organization reported an estimated 10.6 million new tuberculosis (TB) cases globally, with TB deaths reaching 1.6 million.^[1]^ South Korea reported 16,264 TB cases in 2022 and 1356 TB deaths in 2020, ranking TB as the most common notifiable disease in the country. These figures indicate that South Korea has the highest TB incidence and second-highest TB mortality rate among Organization for Economic Co-operation and Development member countries.^[2]^ Additionally, several trends are emerging, including an increasing proportion of elderly patients with TB (aged ≥ 65 years), the occurrence of multidrug-resistant TB cases, and an increase in TB cases imported from abroad.^[2]^

Consistent medication adherence for over 6 months is crucial for successful TB treatment, with nonadherence leading to drug resistance.^[3]^ Noncompliance with this regimen significantly increases the risk of initial treatment failure and progression to multidrug-resistant TB strains.^[3]^ Therefore, self-care capabilities, such as medication adherence, regular hospital visits, and proactive health management throughout treatment, are crucial for patients with TB to avoid treatment failure.^[4]^

Self-care refers to health-related activities undertaken by individuals to meet their functional and developmental needs and maintain a healthy life and functional ability.^[5]^ Self-care performed by patients can maintain or restore their health, minimize illness-induced disabilities, and improve their independence.^[6]^ Therefore, the ability of patients with TB to effectively perform self-care throughout their treatment period is crucial for successful treatment outcomes.

However, self-care among patients with TB in South Korea presents particular challenges, which may be influenced by unique cultural, social, and healthcare system factors in the country.^[7]^ For instance, the stigma associated with TB in Korea is deeply rooted in social perceptions that label TB a poverty-stricken disease that may discourage patients from seeking timely treatment and adhering to long-term medication regimens.^[8]^ Additionally, Korea rapidly aging population contributes to an increased TB burden among the elderly, who may face additional barriers to self-care due to physical frailty, cognitive decline, and a lack of social support.^[9]^ Despite its strengths, the healthcare system in Korea may also pose challenges in terms of accessibility and continuity of care, particularly for vulnerable populations such as the elderly and those with multidrug-resistant TB.^[10]^

A study examining the factors related to self-care in Korean patients with TB investigated their relationships with stigma, stress, depression, self-efficacy, and social support. However, previous research on stigma as a factor influencing self-care in patients with TB has yielded inconsistent results.^[11]^ with some studies indicating that stigma is a major factor, whereas others suggest that it has no effect.^[12,13]^ These inconsistencies may stem from differences in study populations, measurement tools, or cultural contexts, which affect how stigma is perceived and reported.^[13]^ These inconsistencies highlight the need for a more comprehensive analysis. Meta-analysis is an analytical method that can derive more objective research results by statistically analyzing the results of previous studies and considering them as research data.^[14]^

Given the cultural and systemic challenges unique to Korea, a systematic literature review and meta-analysis are needed to comprehensively analyze the factors affecting self-care among Korean patients with TB. This study aimed to address the lack of a comprehensive analysis of self-care factors in Korean patients with TB by determining the effect size of each factor through a systematic review and meta-analysis of the literature.

## 2. Methods

This systematic review and meta-analysis adhered to the Preferred Reporting Items for Systematic Reviews and Meta-Analyses guidelines.^[15]^ Given that the study involved an analysis of the literature and did not involve human subjects, the Institutional Review Board (IRB) of K University granted a formal exemption from review (KNU_IRB_2023-090) as the meta-analysis of existing data was classified as exempt from IRB oversight.

### 2.1. Search strategy

The research question posed was as follows: “What factors are related to self-care in Korean patients with TB?” The PICOS framework (population, intervention, comparison, outcome, and study design) was employed to guide literature selection. The study population was defined as Korean patients with TB; the outcome was self-care, and the study design was correlational. Interventions and comparisons were deemed inapplicable, as the focus was on analyzing the factors related to specific variables.

### 2.2. Inclusion and exclusion criteria

#### 2.2.1. Inclusion criteria

(1) Studies involving Korean patients with TB.
(2) Quantitative studies examining the relationship between patient-perceived self-care and related factors.
(3) Studies that reported Pearson correlation coefficients (*r*) or convertible statistics.
(4) Studies with full texts available.
(5) Studies published since 2000.

#### 2.2.2. Exclusion criteria

(1) Experimental studies, qualitative studies, and reviews.
(2) Studies solely reporting relationships with subscale scores without analyzing the relationship between the total self-care score and related factors.

Studies published before 2000 were excluded to ensure the inclusion of those that reflected the current healthcare context, treatment protocols, and social conditions relevant to TB management in South Korea. The healthcare landscape and treatment strategies for TB have evolved significantly over the past few decades, and studies published before 2000 may not accurately represent contemporary factors influencing self-care in TB patients.

### 2.3. Data extraction

A literature search was performed to identify academic journal articles and master’s and doctoral dissertations published in Korea after January 1, 2000. The data collection period spanned 2 weeks (October 14–28, 2023). Databases such as Koreamed, KMbase, KISS, NDSL (KSITI), National Assembly Library of Korea, and RISS were used. The cited references were manually screened to identify additional studies not indexed in these databases, conducted concurrently with the article screening.

Two researchers independently conducted the search: a researcher with meta-analysis training, and a nursing professor. Key search terms included “tuberculosis” and “self-care,” with “medicine adherence” and “treatment adherence” used as synonymous phrases. These terms were combined and searched using the following search query: “self-care” OR “medicine adherence” OR “treatment adherence” AND “tuberculosis.” The collected papers were organized and saved in Microsoft Excel.

### 2.4. Quality assessment of the studies

Because most of the studies selected for systematic review and meta-analysis were correlational, the Quality Assessment and Validity Tool for Correlational Studies by Cummings et al^[16]^ was used to assess research quality.

### 2.5. Statistical analysis

The data for this study were analyzed using Comprehensive Meta-Analysis software (version 4.0). The effect sizes of the self-care-related factors were calculated and the values were converted from the correlation coefficients (*r*) to Fisher z-values to obtain standardized effect sizes. As the correlation coefficients (*r*) can have either positive or negative values, the direction of the correlation was consistently aligned for interpretation.

A weighted average correlation coefficient was calculated to estimate the overall effect size with weights assigned based on the number of cases in each study. Following Cohen criteria,^[17]^ the effect sizes were classified as small (<0.10), medium (~0.30), or large (≥0.50).

The heterogeneity of the effect sizes was assessed using a forest plot, which visually depicted the direction and confidence intervals of each study’s effect size. The I² statistic, which represents the ratio of true variance to total variance, was used to assess the heterogeneity between studies. Generally, an I² value indicates low heterogeneity at 0% to 25%, moderate heterogeneity at 25% to 75%, and very high heterogeneity at 75% or above.^[14]^ Given the high degree of heterogeneity observed in this analysis (I² = 96.08%), a random-effects model was chosen. The random-effects model is more appropriate in the presence of significant heterogeneity because it accounts for variability both within and between studies, allowing for more generalized conclusions that reflect the diversity of study contexts and populations.

Additionally, publication bias was investigated using Egger regression test and Duval and Tweedie funnel plot analysis.

## 3. Results

### 3.1. Literature selection

**Figure 1.**
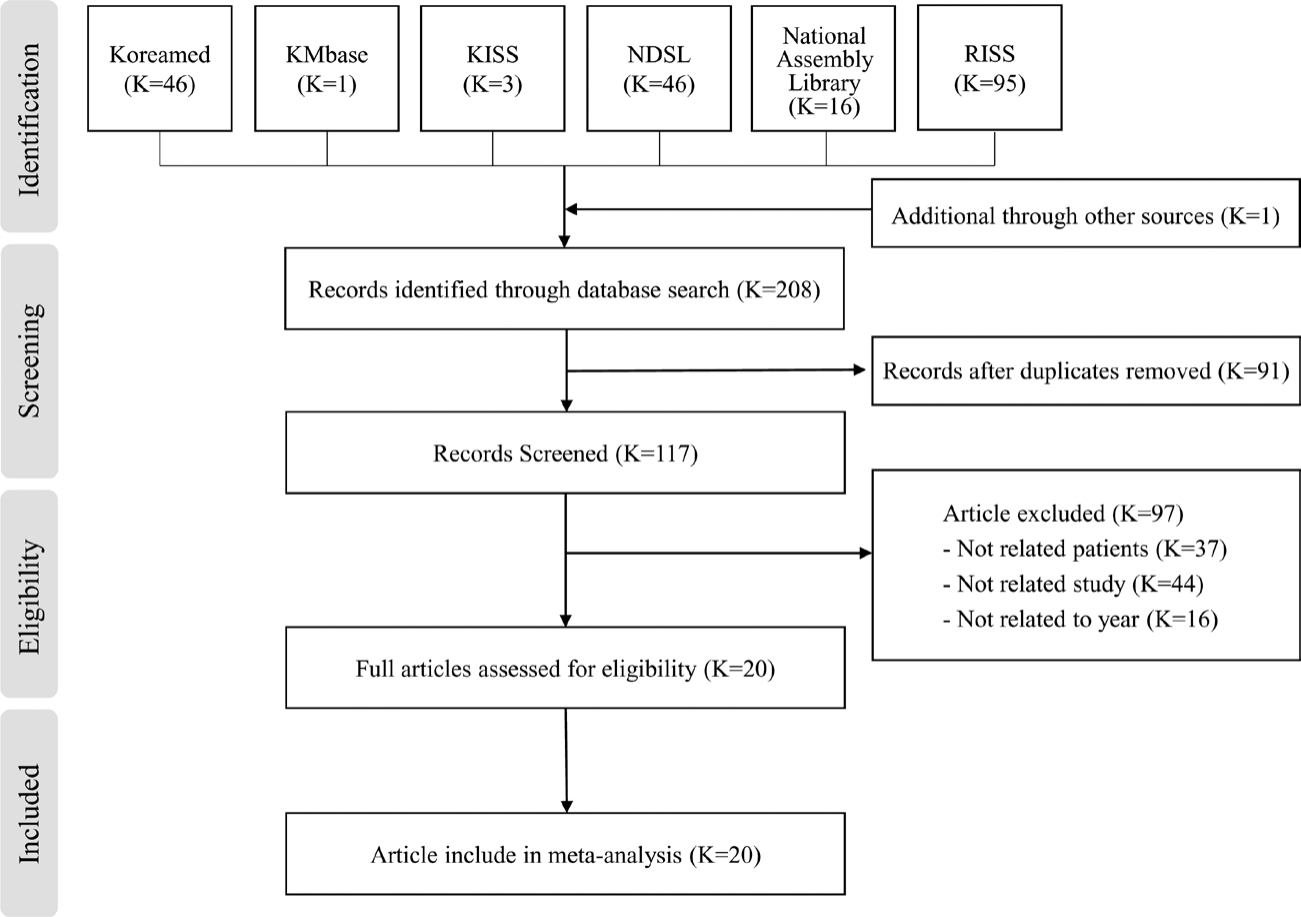
Flow chart of study selection.

Following the literature search, 2 researchers independently verified the number of articles identified. Discrepancies were resolved through discussion and selection of mutually agreed-upon articles. The search yielded 207 articles from databases (Koreamed 46, KMbase 1, KISS 3, NDSL (KSITI) 46, National Assembly Library of Korea 16, and RISS 95) and 1 additional article from the manual search, totaling 208 articles. After removing duplicates (N = 91), 117 titles and abstracts were screened. Of these, 97 articles were excluded because they did not meet the following inclusion criteria: publication year before 2000 (n = 16), outcome unrelated to self-care (n = 44), and population not involving Korean patients with TB (n = 37). Thus, 20 articles were retained for further analysis in this systematic review and meta-analysis (Fig. 1).

### 3.2. Quality assessment of the included studies

The quality of the included studies was independently assessed by 2 researchers. Differences were discussed until consensus was reached regarding the assessment results. Of the 20 studies, 1 study scored 11 points (5%), 5 scored 10 points (25%), 12 scored 9 points (60%), 1 scored 8 points (5%), and 1 scored 6 points (5%). All 20 publications were classified as medium-to high-quality and retained for the final analysis.

### 3.3. Characteristics of the analyzed literature

Of the 20 included studies, 15 (75%) were journal articles and 5 (25%) were dissertations. Participants were recruited from 6 university hospitals, 6 national TB hospitals, 4 general hospitals, and 1 public health center. Two studies recruited participants from both public health centers and hospitals. Six self-care measurement tools were used across the studies: the scale developed by Choi^[18]^ (n = 5), the scale modified by Cho et al^[19]^ from Choi scale (n = 7), the scale modified by Kim et al^[20]^ from Choi tool (n = 3), the Medication Adherence Scale (MMAS-8)^[21]^ (n = 2), Biswas scale^[22]^ (n = 1), and Bae scale^[23]^ (n = 2) (Table 1).

**Table 1:** Descriptive summary of included studies for systemic review (K = 20, N = 2999).

Author (year)	Sample size (n)	Publication	Setting	Participants		Measurements for self-care	Quality assessment
				Age	Gender n (%)		
Jang (2010) [A1]	253	Journal	NTBH	≥20	M:204 (81.6) W:46 (18.4)	CS	9
Sung et al (2011) [A2]	144	Journal	GH	≥18	M:93 (64.6) W:51 (35.4)	CS	10
Cho et al (2013) [A3]	216	Journal	NTBH	All	M:155 (71.8) W:61 (28.2)	CCS	9
Kwon (2013) [A4]	133	Journal	NTBH	All	M:100 (75.2) W:33 (24.8)	CCS	9
Lee et al (2015) [A5]	250	Journal	NTBH	≥20	M:210 (84.0) W:40 (16.0)	CCS	9
Lee et al (2015) [A6]	124	Journal	NDF	15–70	M:59 (47.6) W:65 (52.4)	CCS	6
Yeo (2015) [A7]	163	Thesis	GH	All	M:96 (58.9) W:67 (41.1)	CCS	9
Lee (2015) [A8]	113	Thesis	UH	≥20	M:62 (54.9) W:51 (45.1)	CS	9
Park et al (2015) [A9]	169	Journal	PHC	≥65	M:110 (65.1) W:59 (34.9)	CCS	11
Kim (2016) [A10]	50	Journal	GH	≥20	M:39 (78.0) W:11 (22.0)	CS	8
Jang (2017) [A11]	137	Thesis	PHC, GH	≥18	M:87 (63.5) W:50 (36.5)	MS	10
Ha (2017) [A12]	90	Thesis	NTBH	≥19	M:66 (73.3) W:24 (26.7)	CS	9
Jung et al (2018) [A13]	130	Journal	UH	≥18	M:82 (63.1) W:48 (36.9)	BAS	9
Kim et al (2019) [A14]	140	Journal	UH	≥19	M:77 (55.0) W:63 (45.0)	CKS	9
Lee et al (2020) [A15]	143	Journal	UH	18–65	M:84 (58.7) W:59 (41.3)	BIS	10
Yang et al (2020) [A16]	206	Journal	PHC, GH, UH	≥19	M:73 (35.4) W:133 (64.6)	CKS	10
Park et al (2020) [A17]	125	Journal	NTBH	≥19	M:106 (84.8) W:19 (15.2)	CCS	9
Kim (2021) [A18]	134	Thesis	UH	≥18	M:87 (64.9) W:47 (35.1)	BAS	9
Lee et al (2022) [A19]	119	Journal	UH	≥19	M:68 (57.1) W:51 (42.9)	CKS	9
Jang et al (2022) [A20]	160	Journal	GH	≥18	M:90 (56.2) W:70 (43.8)	MS	10

BAS = Bae scale (2015), BIS = Biswas scale (2014), CS = Choi scale (1983), GH = General hospital, MS = Medication Adherence Scale (MMAS-8), NDF = North Korean defector’s Facility, NTBH = National tuberculosis hospital, PHC = Public health center, UH = University hospital.

### 3.4. Effect sizes of the factors related to self-care

A total of 72 research findings related to self-care were extracted from the 20 included studies. Based on a previous study,^[24]^ which noted that factors with fewer than 2 cases did not significantly contribute to the overall effect size in the meta-analysis, 27 self-care-related factors with fewer than 2 cases were excluded from the analysis, along with 1 factor that could not be statistically processed. The remaining 44 factors were grouped into 5 categories: sociodemographic, TB-related, psychological, environmental, and educational characteristics.

The overall effect size of the 44 factors was significant (ESr = 0.229, 95% CI: 0.112–0.340, *P* < .001). However, there was substantial heterogeneity among the studies (Q = 1096.33 (*P* < .001), I^2^ = 96.08%), indicating a significant variation in individual effect sizes. Therefore, a random-effects model was used in the analysis (Table 2). Among the 5 categories of factors, TB-related characteristics showed a nonsignificant effect size (ESr = −0.376, 95% CI: −0.805 to 0.312, *P* = .281).

**Table 2:** Effect size of sociodemographic and TB-related, psychological, environmental factors related to self-care.

Characteristics	Category	k	ES	95% CI	z	*P*	Heterogeneity				Analyzed model
							Tau^2^	Q	df(p)	I^2^ (p)	
Sociodemographic characteristics	Total	6	−.166	−.459 to .159	−1.00	.317	.160	113.79	5	95.61	Random
	Age	2	.249	.125 to.365	3.88	.000	.000	.245	1	.000	Fixed
	Drinking (yes)	2	−.510	−.849 to .150	−1.54	.124	.248	32.99	1	96.97	Random
	Smoking (yes)	2	−.195	−.296 to −.090	−3.62	.000	.000	.255	1	.613	Fixed
	Comparison between groups	2						30.26	2	(.000)	
TB-related characteristics	Drug discontinuation experience (yes)	3	−.376	−.805 to .312	−1.08	.281	.395	105.09	2	98.10	Random
Psychological characteristics	Total	20	.271	.132 to .400	3.75	.000	.104	341.67	19	94.44	Random
	Health beliefs (sensitivity& severity)	2	.057	−.324 to .423	.286	.775	.076	16.02	1	93.76	Random
	Health beliefs (benefit)	3	.320	.248 to .389	8.31	.000	.003	3.19	2	37.38	Fixed
	Health beliefs (barrier)	3	−.159	−.458 to .171	−.94	.345	.082	35.78	2	94.41	Random
	Stigma	3	.076	−.322 to.451	.365	.715	.122	35.78	2	94.41	Random
	Quality of life	2	.480	.388 to .563	9.02	.000	.005	1.67	1	40.08	Fixed
	Self-efficacy	7	.478	.329 to .603	5.72	.000	.050	47.51	6	87.37	Random
	Comparison between groups	6						21.57	5	(.001)	
Environmental characteristics	Total	13	.450	.362 to .530	9.04	.000	.030	63.82	12	81.20	Random
	Nonfamily support	2	.407	.289 to .514	6.25	.000	.000	.46	1	.000	Fixed
	Family support	8	.382	.283 to .474	7.03	.000	.019	24.31	7	71.20	Random
	Social support	3	.608	.556 to .656	17.49	.000	.001	2.27	2	11.80	Fixed

#### 3.4.1. Sociodemographic characteristics

While the overall effect size of sociodemographic characteristics was not significant (ESr = −.166, 95% CI: −.459 to .159, *P* = .317), analysis of individual factors revealed different patterns: age (ESr = .249, 95% CI: .125 to .365, *P* < .001) and smoking status (ESr = −.195, 95% CI: −.296 to −.090, *P* < .001) showed significant effects, whereas alcohol consumption did not (ESr = −.510, 95% CI: −.849 to .150, *P* = .124). Heterogeneity tests established homogeneity for age and smoking status, allowing the use of a fixed-effects model to present effect sizes. Tests for differences in effect sizes among sociodemographic groups revealed significant differences (Q = 30.26, *P* < .001).

#### 3.4.2. Psychological characteristics

The overall effect size of the psychological characteristics was significant (ESr = .271, 95%CI: .131 to.400, *P* < .001). However, examining individual factors within this category reveals a more nuanced picture. While some subfactors of health beliefs, such as sensitivity, severity, disability, and stigma, did not show significant effects, others, such as perceived benefit (ESr = .320, 95% CI: .248 to .389, *P* < .001), quality of life (ESr = .480, 95% CI: .388 to .563, *P* < .001), and self-efficacy (ESr = .478, 95% CI: .329 to .603, *P* < .001), showed significant positive effect sizes. Heterogeneity tests indicated significant variation among the studies for self-efficacy (I^2^ = 87.37), necessitating a random-effects model for analysis. In contrast, perceived benefits and quality of life exhibited homogeneity, allowing the use of a fixed-effects model. Finally, a significant difference in the effect size was found across the 6 psychological characteristic groups (Q = 21.57, *P* < .001).

#### 3.4.3. Environmental characteristics

The overall effect size of the environmental characteristics was significant (ESr = 0.450, 95% CI: 0.362–0.530, *P* < .001). Further analysis of individual environmental factors revealed that social support had the largest effect size (ESr = 0.608, 95% CI: 0.556–0.656, *P* < .001), followed by nonfamily support (ESr = 0.407, 95% CI: 0.289–0.514, *P* < .001) and family support (ESr = 0.382, 95% CI: 0.283–0.474, *P* < .001). Heterogeneity tests indicated that fixed-effects models were appropriate for nonfamily and social support, whereas a random-effects model was chosen for family support because of the presence of significant heterogeneity (Q = 22.63, *P* < .001).

#### 3.4.4. Educational characteristics

The category “educational characteristics” had only 1 factor: TB-related knowledge. This factor had a significant positive effect on self-care (ESr = .284, 95% CI: .183–.378, *P* < .001). The heterogeneity test indicated homogeneity for TB-related knowledge, justifying the use of a fixed-effects model to determine the effect size.

### 3.5. Publication-bias test

**Figure 2.**
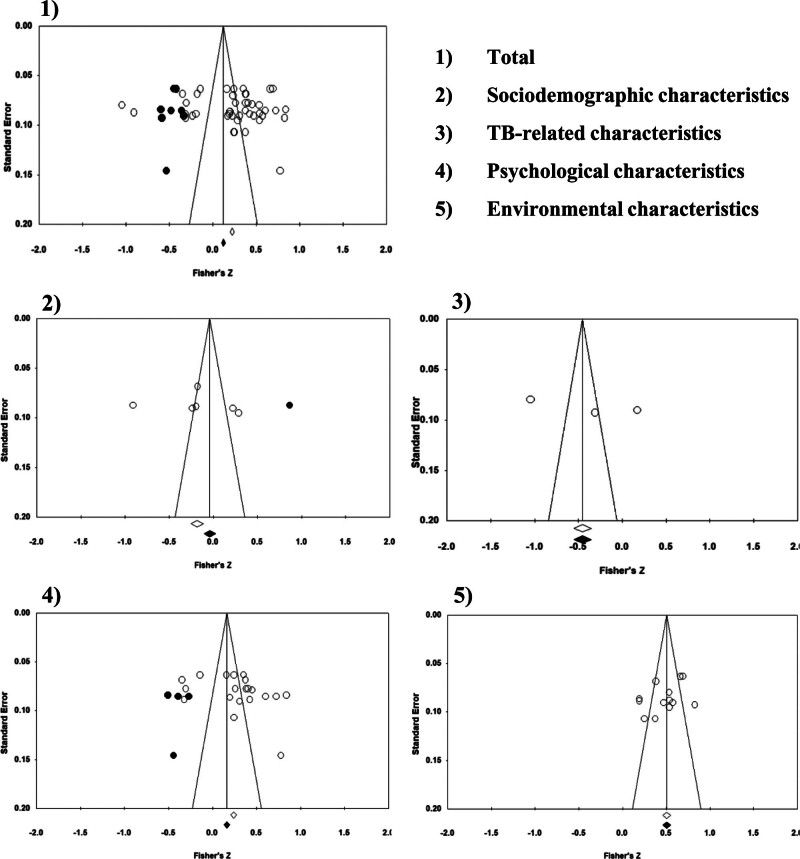
Funnel plot of publication bias.

Publication bias was assessed using Egger regression intercept and significance test and Duval and Tweedie funnel plot, with the addition and removal of studies for symmetry assessment. The adjusted effect sizes were presented based on these analyses. Studies that were added or removed based on the Duval and Tweedie funnel plot analysis are marked in black in Figure 2. Egger regression analysis revealed no significant intercepts across all groups, indicating the absence of overall publication bias. Duval and Tweedie funnel plot symmetry test revealed that overall group symmetry could be achieved with the addition of 8 studies on the left side of the funnel plot, which would reduce the effect size from .229 to .126 (Table 3).

**Table 3:** Publication bias.

Category	k	Egger regression intercept		Duval and Tweedie trim and fill				
		Intercept	*P* value	Model	Studied trimmed	Estimate	Lower	Upper
Total	44	1.356	.774	Observed	–	.229	.112	.340
				Adjusted	8	.126	.004	.244
Sociodemographic characteristics	6	6.612	.740	Observed	–	−.166	−.458	.159
				Adjusted	1	−.049	−.397	.364
TB-related characteristics	3	76.089	.333	Observed	−	−.376	−.805	.312
				Adjusted	0	−.376	−.805	.312
Psychological characteristics	20	7.904	.150	Observed	−	.271	.132	.400
				Adjusted	4	.167	.017	.309
Educational characteristics	13	−5.264	.165	Observed	−	.450	.263	.530
				Adjusted	0	.450	.263	.530

In the subgroup analysis, the educational characteristics category was excluded because it had fewer than 3 cases. Symmetry was achieved for TB-related and environmental characteristics, indicating no publication bias in these factor categories. However, subgroup comparisons of other factor categories revealed a potential bias. Therefore, the overall effect size of sociodemographic characteristics was adjusted from −.166 to −.019 with the addition of 1 study on the right, and the effect size of psychological characteristics was adjusted from .271 to .167 with the addition of 4 studies on the left (Table 3).

### 3.6. Influence of study quality on findings

Subgroup analysis revealed that study quality significantly influenced the findings. High-quality studies (scoring 9 or 10 points) generally reported larger effect sizes for psychological factors, such as self-efficacy and quality of life, as well as for environmental factors, such as social support, likely because of the more robust methodologies and better control of confounding variables. By contrast, lower-quality studies tended to report smaller or less significant effect sizes, particularly for sociodemographic factors. These findings highlight the critical role of methodological rigor in accurately assessing factors related to self-care in TB patients. Future research should prioritize the maintenance of high-quality standards to obtain more reliable results.

## 4. Discussion

This study conducted a systematic review and meta-analysis to investigate the relationship between various factors and self-care in Korean patients with TB. The analysis revealed that, while TB and sociodemographic factors did not have a significant impact, environmental, educational, and psychological factors significantly influenced self-care, with environmental factors having the most substantial effect.

The lack of significance of TB-related and sociodemographic factors can be attributed to several methodological and contextual factors. Methodologically, the definitions and measurements of these factors varied across studies, potentially diluting their collective impact.^[25]^ For example, differences in TB severity, the presence of comorbidities, and variations in treatment regimens may have contributed to these inconsistencies.^[26]^ Contextually, the evolution of TB treatment and management strategies over time could mean that factors previously considered important may no longer be as impactful due to advancements in care practices and medications.^[1]^ Additionally, in South Korea, the high burden of TB combined with universal healthcare coverage may mitigate the influence of sociodemographic factors such as income or education level on self-care practices.^[27]^

The strong effect of environmental factors, particularly social support, highlights the importance of support networks in managing chronic conditions, such as TB. Social support from family, friends, healthcare professionals, and the community plays a crucial role in enhancing self-care and treatment adherence.^[28]^ Psychological factors, such as self-efficacy and quality of life, are also vital in effectively managing treatment regimens. Patients with higher self-efficacy are more likely to engage in health-promoting behaviors such as medication adherence, regular exercise, and maintaining a healthy diet.^[29]^ Educational factors such as TB-related knowledge empower patients to actively participate in their care.

Therefore, future self-care strategies for TB should prioritize addressing the environmental, educational, and psychological factors. Strengthening social support and building community networks is essential. For instance, establishing peer support groups where TB patients can share experiences and encourage one another can reduce feelings of isolation and increase motivation to adhere to treatment protocols.^[30]^ Additionally, educational programs aimed at enhancing self-efficacy and TB-related knowledge should be emphasized, including workshops and counseling sessions focused on goal setting, problem-solving, and stress management. Healthcare providers should also be trained to recognize and address psychological barriers to treatment such as stigma and depression.

Although publication bias was assessed using Egger regression intercept and funnel plots, the possibility of residual bias cannot be entirely ruled out. For example, unpublished studies or studies in languages other than Korean might have influenced the results if they were not captured in the search process.^[31]^ Additionally, excluding studies published before 2000 and non-Korean patient studies might introduce bias. Older studies may have different findings due to changes in treatment protocols and healthcare practices over time, and non-Korean studies could provide insights into how different cultural or healthcare system factors affect self-care.^[32]^

This study’s findings have implications beyond the Korean context. TB patients in other countries with similar healthcare challenges or cultural factors may experience similar issues. In countries such as India and parts of Africa, where TB incidence is high and stigma plays a significant role,^[33,34]^ psychological and environmental factors may also be crucial in self-care. In this context, addressing stigma, strengthening social support networks, and improving patient education are important. Moreover, insights from this study could inform global TB self-care strategies by emphasizing the need for culturally and contextually relevant interventions that address the psychological and environmental factors.

## 5. Conclusion

This study provides a comprehensive analysis of the factors influencing self-care practices among Korean patients with TB through a systematic review and meta-analysis. The findings revealed that, while TB-related and sociodemographic factors did not significantly impact self-care, environmental, educational, and psychological factors played a crucial role, with environmental factors, particularly social support, having the most substantial effect.

These results suggest that effective TB self-care strategies should prioritize interventions that address these key areas. Strengthening social support networks, enhancing patient education, and improving psychological resilience are critical components that can significantly improve self-care adherence among TB patients. Incorporating community-based support programs and tailored educational workshops into existing healthcare frameworks could lead to better treatment adherence and overall outcomes. This study also highlighted the potential for similar approaches to be applied in other countries with comparable healthcare challenges or cultural factors, suggesting that focusing on environmental and psychological support can enhance global TB self-care.

Furthermore, this study differs from previous studies in that it provides a more nuanced understanding of the factors that strongly influence self-care. While previous studies have explored the role of individual factors, such as stigma or self-efficacy in isolation, this meta-analysis synthesizes a broader range of influences, offering a more holistic view that can inform more targeted and effective interventions.

However, this study has some limitations, including potential publication bias and the exclusion of older studies and non-Korean patient studies, which may have influenced the findings. Therefore, ongoing research is needed to build upon these results to ensure that TB self-care strategies remain adaptable and effective in the face of the evolving healthcare landscape. Future studies should also explore the practical implementation of these findings in clinical settings and evaluate their impact on patient outcomes to further refine the TB self-care strategies.

## Acknowledgments

The authors would like to thank Byung-Ryong Ahn, PhD for his assistance with statistical analysis. This study was conducted with approval for exemption from deliberation by the Institutional Ethics Review Committee of K University (KNU_IRB_2023_090). The protocols used in this study were not registered.

## Author contributions

**Conceptualization:** Go Un Lee.

**Data curation:** Hye Kyung Lee, Go Un Lee.

**Formal analysis:** Hye Kyung Lee, Go Un Lee.

**Funding acquisition:** Hye Kyung Lee.

**Investigation:** Hye Kyung Lee, Go Un Lee.

**Methodology:** Hye Kyung Lee, Go Un Lee.

**Project administration:** Hye Kyung Lee, Go Un Lee.

**Resources:** Hye Kyung Lee, Go Un Lee.

**Software:** Hye Kyung Lee, Go Un Lee.

**Supervision:** Hye Kyung Lee.

**Validation:** Hye Kyung Lee, Go Un Lee.

**Visualization:** Hye Kyung Lee, Go Un Lee.

**Writing – original draft:** Go Un Lee.

**Writing – review & editing:** Hye Kyung Lee, Go Un Lee.
